# Alterations of ribosomal RNA pseudouridylation in human breast cancer

**DOI:** 10.1093/narcan/zcad026

**Published:** 2023-05-30

**Authors:** Chiara Barozzi, Federico Zacchini, Angelo Gianluca Corradini, Monica Morara, Margherita Serra, Veronica De Sanctis, Roberto Bertorelli, Erik Dassi, Lorenzo Montanaro

**Affiliations:** Department of Medical and Surgical Sciences (DIMEC), Alma Mater Studiorum - University of Bologna, Bologna I-40138, Italy; Centre for Applied Biomedical Research – CRBA, University of Bologna, Sant’Orsola Hospital, Bologna I-40138, Italy; Department of Medical and Surgical Sciences (DIMEC), Alma Mater Studiorum - University of Bologna, Bologna I-40138, Italy; Centre for Applied Biomedical Research – CRBA, University of Bologna, Sant’Orsola Hospital, Bologna I-40138, Italy; Unit of Pathology, IRCCS Azienda Ospedaliero-Universitaria di Bologna, Via Albertoni 15, I-40138 Bologna, Italy; Department of Medical and Surgical Sciences (DIMEC), Alma Mater Studiorum - University of Bologna, Bologna I-40138, Italy; Departmental Program in Laboratory Medicine, IRCCS Azienda Ospedaliero-Universitaria di Bologna, Via Albertoni 15, I-40138 Bologna, Italy; Unit of Breast Surgery, IRCCS Azienda Ospedaliero-Universitaria di Bologna, Via Albertoni 15, I-40138 Bologna, Italy; Department of Cellular, Computational and Integrative Biology - CIBIO, University of Trento, Povo (TN) I-38123, Italy; Department of Cellular, Computational and Integrative Biology - CIBIO, University of Trento, Povo (TN) I-38123, Italy; Department of Cellular, Computational and Integrative Biology - CIBIO, University of Trento, Povo (TN) I-38123, Italy; Department of Medical and Surgical Sciences (DIMEC), Alma Mater Studiorum - University of Bologna, Bologna I-40138, Italy; Departmental Program in Laboratory Medicine, IRCCS Azienda Ospedaliero-Universitaria di Bologna, Via Albertoni 15, I-40138 Bologna, Italy

## Abstract

RNA modifications are key regulatory factors for several biological and pathological processes. They are abundantly represented on ribosomal RNA (rRNA), where they contribute to regulate ribosomal function in mRNA translation. Altered RNA modification pathways have been linked to tumorigenesis as well as to other human diseases. In this study we quantitatively evaluated the site-specific pseudouridylation pattern in rRNA in breast cancer samples exploiting the RBS-Seq technique involving RNA bisulfite treatment coupled with a new NGS approach. We found a wide variability among patients at different sites. The most dysregulated positions in tumors turned out to be hypermodified with respect to a reference RNA. As for 2′O-methylation level of rRNA modification, we detected variable and stable pseudouridine sites, with the most stable sites being the most evolutionary conserved. We also observed that pseudouridylation levels at specific sites are related to some clinical and bio-pathological tumor features and they are able to distinguish different patient clusters. This study is the first example of the contribution that newly available high-throughput approaches for site specific pseudouridine detection can provide to the understanding of the intrinsic ribosomal changes occurring in human tumors.

## Graphical Abstract

**Graphical Abstract:**
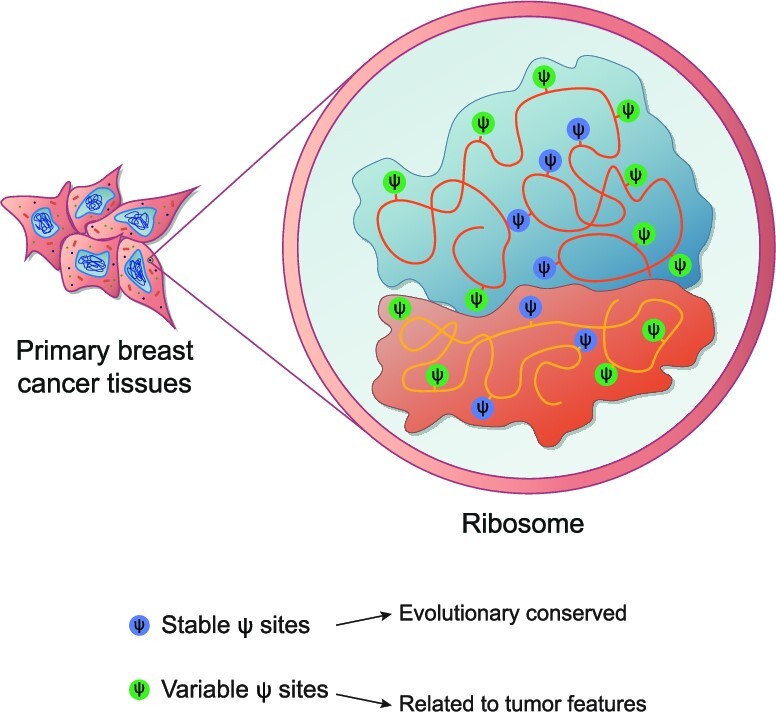

## INTRODUCTION

Alterations in ribosome biogenesis and ribosomal function are constant features of cancer cells (1–3). Such changes also include those leading to intrinsic ribosomal alterations involving ribosomal proteins and ribosomal RNA (rRNA) (4–9). The majority of intrinsic ribosomal changes affecting rRNA are related to an altered RNA modification (9–11). RNA modifications are emerging as pivotal cellular regulators, not only for direct functional effects on gene expression but also for several biological processes and tissue development, as well as for development of diseases. RNA modifications are abundantly represented on rRNA, where they contribute to the stabilisation of the secondary and tertiary structure of the rRNA itself, thus ensuring the efficiency and accuracy of translation. Furthermore, these modifications may also modulate the activity of ribosomes in response to environmental changes, growth signals, and in pathological conditions (12).

Of the 228 RNA modifications discovered so far (13) in human 80S ribosomes, the majority are represented by 2′-O-methylation of ribose (112 known sites), which can occur at any nucleotide, and the conversion of uridine into its 5-ribosyl isomer, pseudouridine (Ψ), resulting in the addition of an extra carbon–carbon bond between the base and the sugar, and a hydrogen bond donor. The first studies in yeast revealed that some 2′-O-methylation sites are critical for ribosomal functions and cell survival, while the loss of other sites has little or no effect (14). The development of high-throughput techniques such as RiboMethSeq allowed the identification of variable ribomethylation levels across the modification sites in cell line models of human malignancies (15). Recently, the profiling of the first two clinical cohorts of tissue samples from breast cancer and B-cell lymphoma patients further confirmed the co-existence of stable sites, showing limited invariability in their 2′-O-methylation level, together with variable sites of 2′-O-methylation which are highly likely to undergo specific regulation during normal and pathological processes. These results support the plasticity of the ribosome structure, impacting translational regulation in humans, including in cancer (11,16).

In rRNA, pseudouridylation is carried out in a site-specific fashion by ribonucleoprotein (RNP) complexes called H/ACAbox RNPs, each consisting of one H/ACA snoRNA and four core proteins, namely GAR1, NHP2, NOP10 and dyskerin (DKC1). H/ACA snoRNAs are small non-coding RNAs of 60–300 nucleotides (nts), containing a secondary structure called hairpin-hinge-hairpin-tail, which harbours a pseudouridylation pocket specific to a particular target sequence, based on sequence‐specific base pairing. The interaction between the guide snoRNA and the substrate guides the enzymatic complex on the target uridine, which is subsequently isomerized into Ψ by DKC1, the component of the complex endowed with pseudouridine synthase activity (reviewed in (17–19). On one side pseudouridines are known to confer increased rigidity to the phosphodiester backbone of the RNA through effects on base stacking and water coordination. In addition, they are crucial for the formation of specific tertiary structures which may depend on their specific location (stem vs loop) and sequence context (20–22). Pseudouridine can also confer local conformational dynamics which play important roles in ribosome assembly and maintenance of the ribosomal structural integrity during translation. Moreover, pseudouridylation at specific sites can facilitate the further modification of adjacent nucleotides by altering the allosteric arrangement of rRNA (22).

Although the isomerization of uridine has been widely studied, little is known about the effects of dysregulation of pseudouridylation on rRNA, primarily due to technical limitations in site-specific pseudouridine detection (23). This was firstly accomplished by CMC treatment followed by alkaline hydrolysis, which selectively and irreversibly binds to pseudouridine leading to premature truncation of retrotranscription at the modified site. Recently, this approach has been combined to massive parallel sequencing which revealed the high dynamism and functional importance of pseudouridylation in different environmental conditions, growth state, and tissue-specificity (12,24–26). On the other hand, CMC-based methods have some limitations, primarily false negatives, likely caused by not perfectly efficient conjugation of CMC to Ψ, and false positives, due to ineffective alkaline hydrolysis of CMC from guanosine and uracil. These issues limit the quantitative assessments of pseudouridylation and are responsible for the lack of consistency in the different datasets. Thanks to a method for quantitative RNA analysis based on liquid chromatography-mass spectrometry (LC-MS) technology, all the RNA modifications of the human 80S ribosome were recognized and located in its three-dimensional structure (13). This study showed that all modification sites reside in functionally essential interior regions, including the catalytic centre, the A, P and E sites for tRNA and mRNA binding, expanding to the exterior regions in humans, where the modifications stabilize the ribosomal structure thanks to interactions with specific proteins. Such an approach, although exhaustive at the atomic resolution, lacks high-throughput potential for a wide series investigation. Recently, an innovative method called RNA Bisulfite Sequencing (RBS-Seq) (27) was developed for the simultaneous profiling of m^5^C, m^1^A and Ψ sites in the transcriptome. Regarding Ψ, bisulfite forms a stable adduct on the 1′ carbon of ribose, causing the opening of the ribose ring. During cDNA synthesis for library preparation, the Ψ-bisulfite adduct forces the reverse transcriptase to skip the base leading to a typical deletion signature at the modified sites in the sequencing output. This technique proved to be quantitative and site-specific, both for coding and non-coding RNAs, shedding new light on the human epitranscriptome (27).

Alterations in the expression levels of RNA modifiers and thereby, dysregulated RNA modification pathways, have been linked to tumorigenesis as well as to other human diseases (28). In breast cancer cell lines, the downregulation of DKC1 was found to be related to reduced global rRNA pseudouridylation (10) while in breast carcinomas DKC1 expression is strictly associated with survival. Tumors displaying low levels of DKC1 are indeed characterized by a more favourable clinical outcome (10). Furthermore, DKC1 overexpression supported the neoplastic transformation of mammary epithelium in terms of increased invasive, staminal and clonogenic potentials, and increased ribosome efficiency together with a remodulation in snoRNAs expression levels (29). These results were confirmed by another study on breast cancer, which highlighted the significant association between high expression of DKC1, both at the mRNA and protein level, and clinical–pathological parameters, poor prognosis and short survival (30).

Similarly, in many neoplastic conditions, small nucleolar RNAs (snoRNAs) are frequently altered (31). For instance, SNORA3, SNORA18, SNORA7B, SNORA13 and SNORA2A are overexpressed in aggressive breast cancer while SNORA15 and SNORA24 appear to be downregulated in haematological neoplastic diseases. Therefore, dysregulation of specific H/ACA snoRNAs may directly affect ribosomal biogenesis and function by altering the specific pattern of rRNA pseudouridylation. Hence, alterations in H/ACA snoRNA expression and in the pseudouridylation pattern may affect rRNA biogenesis, protein synthesis and cell growth, suggesting that they contribute to ribosomal function in a synergistic way. The involvement of both pseudouridylation and snoRNA expression has not been investigated yet in breast cancer comprehensively. Therefore, the aim of the present study is the quantitative and site-specific evaluation of the pseudouridylation profile of rRNA in a series of breast carcinomas by the RBS-Seq technique. In addition, we investigate the association of the pseudouridine site dysregulation with bio-pathological features of our breast cancer series to assess its clinical relevance. Eventually, we analyze the co-occurrence of alterations in the expression of H/ACA box snoRNA guiding the Ψ modification in the same tumor. We therefore provide new potential leads to exploit pseudouridine-modified rRNA sites for the development of therapeutic approaches in breast cancer.

## MATERIALS AND METHODS

### Human samples and RNA preparation

Breast cancer samples were prospectively collected from 2019 to 2021 after surgical resection and written informed consent of patients not undergoing neoadjuvant chemotherapy. The study (SNORA-CaM—140/2019/Sper/AOUbo) was approved by the local Ethical Committee. RNA preparation and subsequent bisulfite treatment and RNA recovery were carried out as reported by Khoddami et al. (27) with some modification since we adapted the RBS-Seq method for investigating ribosomal RNA.

Total RNA was extracted from frozen specimens of 34 breast tumors using the mirVana miRNA isolation kit (ThermoFisher Scientific). Human total RNA (Human XpressRef Universal Total RNA, Qiagen) was used as normal reference samples for analysis as in Marcel *et al.* (11). Five μg of total RNA were submitted to DNAse digestion and chemical fragmentation (see Supplementary Material) prior to bisulfite treatment, while for the non-treated samples 500 ng were used. For each tumor and reference sample, a bisulfite treatment (RBS sample) and a non-treated fragmented sample (NBS sample) were processed in subsequent experiments.

Two sequencing runs were planned. The first one included 11 samples in duplicate with the corresponding non-treated sample, while the second one included 22 RBS samples in single, 4 replicates of the RNA reference sample and all the corresponding NBS samples.

### Bisulfite treatment, library preparation and sequencing

Bisulfite treatment and RNA recovery were carried out as reported by Khoddami *et al.* (27). Briefly, samples were incubated with 312 μl of freshly prepared 5M sodium bisulfite (pH 5) and 3 μl of freshly prepared 100mM hydroquinone at 50°C, rotating for 16 h. Desulfonation was performed adding 0.5 ml of 2M Tris buffer pH 9.0 at 37°C for 2 h. After ethanol precipitation, samples were resuspended in 20 mM MgCl2 and 50 mM Tris–HCl pH 7 and incubated at 75°C for 15′ to finalize the adduction of monobisulfite to the Ψ nucleoside (see Supplementary Material for details). Bisulfite treated samples and only fragmented non-treated samples were purified and size-selected (with fragments ranging from 25 to about 200–220 nts) using RNAClean XP paramagnetic (Agencourt) beads according to a modified manufacturers’ protocol (as in Supplementary Material).

RNA library construction was performed with the TruSeq Stranded mRNA kit (Illumina) with some modifications. In particular, 100 ng of each beads-purified aqueous RNA samples were submitted to a second round of RNACleanXP beads purification to substitute water with the FPF (Fragment, Prime, Finish Mix) solution included in the library kit. Then the manufacturer's protocol was followed. RNA fragments were reverse transcribed using random primers and the addition of Actinomycin D preventing spurious DNA-dependent synthesis, while strand specificity was achieved by replacing dTTP with dUTP in the second strand cDNA synthesis step. After 3′ ends adenylation and adapter ligation, products were enriched with PCR using a Polymerase which does not incorporate past dUTP. Enriched products were size-purified (150–350 bp) by paramagnetic beads (AMPure XP Reagent, Beckman Coulter) to create the final cDNA library. In the adapter ligation step, the unique-dual indexes (Illumina) were used. Each individual library was then quantified and quality-controlled using Qubit Fluorometer (ThermoFisher Scientific) and LabChip GX (Perkin Elmer). An unbalanced pooling design was used to achieve a ratio of 1–8 between untreated and BS-treated samples. After libraries unbalanced pooling, the final pool was quality checked again with Qubit, LabChip GX, and qPCR (KAPA and BIORAD). The adaptor-tagged pool of libraries was loaded on 1 Illumina Novaseq6000 S1 flow-cell for cluster generation and deep sequencing with PE100 chemistry. This sequencing platform is available at the CIBIO NGS facility at the University of Trento.

### H/ACA box snoRNAs expression by qRT-PCR

Total RNA of breast cancer specimens used for RBS-seq was subsequently reverse-transcribed using the GOScript kit (Promega) at 55°C following the manufacturer's protocol. Then quantitative real-time PCR (qRT-PCR) with sybr green was assessed to determine the expression of the following H/ACA box snoRNAs: SNORA5A-B; SNORA5C, SNORA22A-B-C; SNORA33, SNORA41, SNORA61, SNORA64, SNORA67, SNORA70 (detailed in Supplementary Material; primers are listed in Table S1).

### Bioinformatic and statistical analysis

Reads filtering, adapter trimming and alignment of the reads are detailed in Supplementary Material. rRNA was aligned to corresponding reference sequence from the NCBI (https://www.ncbi.nlm.nih.gov/; #U13369.1).

The bisulfite-converted (RBS) and untreated samples (NBS) were then used as input for the ScorePseudouridinePosition tool of the RBSSeqTools v1.0 suite (27).

For each RBS sample, the corresponding NBS sample was used as the non-bisulfite sample, using a threshold on the fraction of deletion reads at each position in the NBS sample of 10%. The per-position quantification and filtering produced by ScorePseudouridinePosition was used to analyze the fraction of modification of known modified positions on rRNAs, according to a list of such position obtained by Taoka *et al.* (13) accounting for a total of 106 positions in 5.8S, 18S and 28S rRNA. The first step of the analysis on RBS samples consisted in filtering out positions not satisfying the following criteria: (a) at least 10 reads in both RBS and NBS samples; (b) at least 5 deletions in RBS; (c) at least 5% of the reads with deletion on RBS samples; (d) the percentage of deletion in NBS less than the threshold of 10%.

For each of these positions, a binomial test *P*-value test was computed on the fraction of reads with deletion in both bisulfite-converted and untreated samples, using an expected ratio of deletion of 1%. Positions significant (BH-adjusted *P* ≤ 0.05) in the RBS but not in the corresponding NBS samples were deemed significant and considered for further analyses. Eventually, we performed a binomial test on the fraction of reads with deletion for significant positions, comparing each RBS sample with a pool of four normal reference samples (whose average ratio of deletion reads was used as expected ratio). Significant positions (BH-adjusted *P* ≤ 0.05, having therefore a significantly increased/decreased ratio of deletion with respect to reference samples) were considered for further analyses. The log_2_ fold-change (FC), obtained by comparing the deletion rate of significant position in RBS samples to the reference sample average, was eventually used for downstream analyses and graphical representation. Principal component analysis and k-means clustering were performed with R v 4.1 (https://www.R-project.org/). Differences in clinical parameters between the identified clusters were statistically evaluated by the Wilcoxon rank-sum test (continuous variables) and Fisher's exact test (categorical variables).

## RESULTS

In the present study, we quantitatively evaluated the site-specific rRNA pseudouridylation in breast cancer samples and its relationship with tumor clinical and biopathological features. For this purpose, we adapted to rRNA the RBS-seq method described by Khoddami *et al.* for the discovery of novel Ψ sites in mRNA and developed a dedicated pipeline to detect any variation in the modification levels of known rRNA pseudouridines in tumors with respect to normal control samples. From the analysis of the data we obtained, for each sample, a typical deletion rate corresponding to a percentage of Ψ-modification for each known position on rRNA.

### RBS-seq reproducibility and coverage on rRNA from breast cancer samples

Since RBS-Seq has never been performed on rRNA from human tumor samples, we aimed to assess the reproducibility of the technique by evaluating the concordance of 4 technical replicates for the RNA reference and 2 replicates from 11 samples. The correlation matrix obtained by the Pearson test indicated a high level of correlation, with *r* ≥ 0.95 in all but one sample (Supplementary Figure S1). All additional samples were therefore evaluated without replicates.

In total, we sequenced 34 breast cancer specimens and obtained a read depth in the order of millions, both for RBS and NBS samples (mean 66857995 ± 21089401, min 32223210, max 108908436; 14290918 ± 3493731, min 8572738, max 19182958, respectively) with all RBS samples having more than 30 million reads. This result is consistent with the experimental design which aimed at a read depth high enough to call the protocol-induced deletions with good accuracy and sensitivity. Reads were then mapped on the three components of rRNA which are pseudouridylated, namely 18S, 28S and 5.8S. All samples reached at least 50% of reads mapped on these sequences, with the minimum value obtained in sample 23A (52.24%). However, an imbalance between RBS and NBS data is evident, with NBS samples characterised by a higher percentage of mapping reads. This could be explained by the effect of sodium bisulfite treatment, which may strongly damage RNA which is rich in modifications and hence prone to cleavage in these sites. Normalising the number of mapped reads for the respective sequence length, i.e. 1869 nt for the 18S, 5035 nt for the 28S and 157 nt for the 5.8S, shows that the mapped reads per base are mostly homogeneous within the 2 groups of samples (NBS and RBS, Supplementary Figure S2). Moreover, the bisulfite treatment of smaller RNA fragments (such as e.g. 5.8S rRNA) can in principle cause the degradation and consequent loss of material in the following size selection. In line with this we observed a limited coverage of 5.8S sequences in comparison to other rRNA sequences (Supplementary Figure S2). Finally, to inspect whether the signal obtained by the bisulfite treatment was even along the length of 18S and 28S rRNAs we checked the modification fraction obtained by the average of the reference sample (Supplementary Figure S3A, B). By this analysis, we find that the modification signal is variable but not dependent on the position in the RNA sequence.

### Evaluation of rRNA pseudouridylation levels in primary breast cancer samples

We then assessed the deletion rate in each breast cancer sample at each known Ψ position defined by the mass spectrometry study on rRNA modification performed by Taoka *et al.* (13). As expected we observed a much stronger deletion signal at the known pseudouridylation sites compared to other positions in rRNA with a progressively decreasing smear of the deletion signal in the positions adjacent to the known modification sites (Supplementary Figure S4). Overall, pseudouridylation levels resulted to be highly variable both within positions and samples, with modification percentages ranging between 5% and 99% (Supplementary Table S2). After the first filtering step, three positions were excluded in all considered cases due to undetectable levels of modification, namely Ψ296 on 18S rRNA and Ψ55 and 69 on 5.8S rRNA. Positions 55 and 69 on 5.8S, the only two known sites of pseudouridylation in this rRNA, are probably lost as a consequence of the harsh conditions of bisulfite treatment on the small 5.8S rRNA fragment.

**Figure 1.**
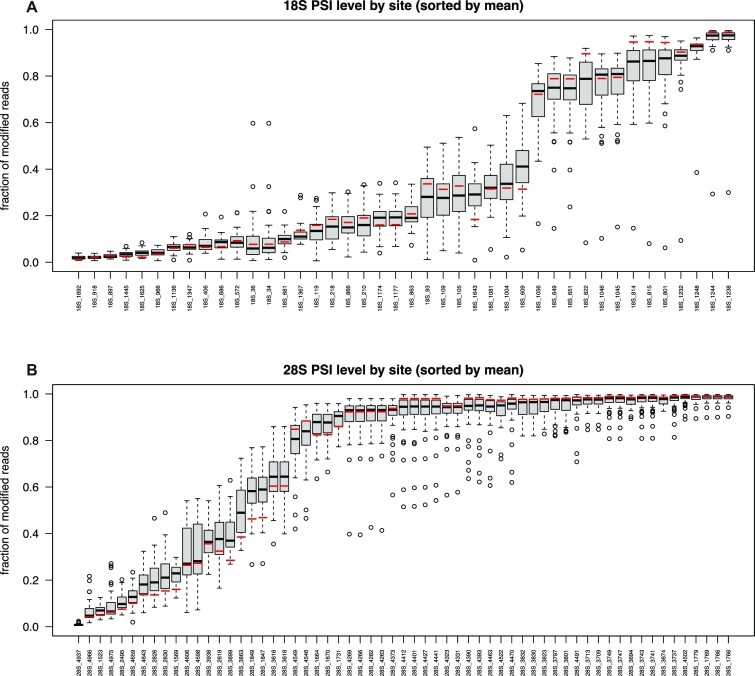
Box plot representing the modification fraction of all samples and the mean of reference RNA samples (red bar) ranked by increasing average value (black bar) of modified uridines in 18S (**A**) and 28S (**B**) rRNA. Interquartile range (black box) and outliers (black circles) are also depicted to show inter-patients variability.

Levels of pseudouridine modification of breast cancer samples, together with reference RNA ones, were ranked by increasing average fraction of modified uridines (Figure 1A, B). Interestingly, in both 18S and 28S rRNA we observed a wide variability among sites displaying an intermediate level of modification, with the highly and lowly modified positions considerably less variable across samples. After reads filtering, positions 1248 (reported to be a m^1^acp^3^Ψ) on 18S and 4937 on 28S were excluded in some samples as showing a deletion fraction >10% in the NBS or <5% in RBS sample.

We then performed a statistical evaluation, by a BH-corrected binomial test, of the observed modification levels in RBS samples with respect to NBS, allowing the removal of technical artefacts in Ψ detection. Consequently, Ψ1248 was excluded from all samples, while Ψ4937 was excluded from 29 samples. Some additional positions (11 out of 108, 10%) were also excluded in at least one patient. Supplementary Table S3 reports all excluded positions. In one sample (S17) most positions (98/108) were excluded after filtering. That sample was therefore not further considered in our analysis. Position 4500 on 28S rRNA, which is a m^3^U, was never detected in our samples, supporting the robustness of our analysis. Finally, we evaluated the statistical significance of the modification levels found in tumors compared to RNA reference samples by determining the log_2_ fold-change (log2FC) between the modification rate of the samples compared to reference RNA. As regards samples in duplicate and the 4 replicates of the reference RNA, the arithmetic mean was used for the calculation of the FC. The obtained results are shown in the 18S and 28S heatmaps, with Ψ positions clustered by their log_2_FC across samples (Supplementary Figure S5A, B).

**Figure 2.**
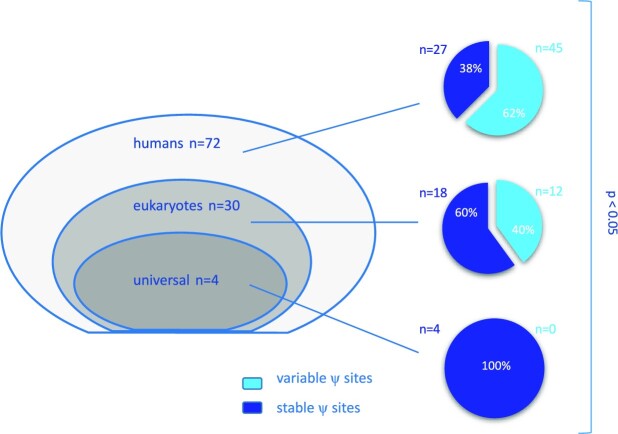
Evolutionary conservation of pseudouridine sites in rRNA: among 106 positions, 72 are specific to humans, 30 are found across eukaryotes and 4 are conserved from bacteria to humans and are referred to as ‘universal’ (left panel). On the right panel, pie charts showing the proportion of ‘variable’ and ‘stable’ sites evaluated in our series of breast cancers according to the three groups. *P* < 0.05 by chi-square test.

We then evaluated pseudouridylation sites variability on the basis of evolutionary conservation according to the 3D Ribosomal Modification Maps database and Taoka *et al.* (13). In these sources, 72 Ψ sites are defined as specific to humans, 30 are found across eukaryotes and 4 are conserved from bacteria to humans and are referred to as ‘universal’. For each position, we defined the samples significantly differing from the reference RNA; positions were considered ‘variable’ if significantly different from reference RNA in more than 11 samples out of 33 (33% of total cases). We observed an increasing variability in the pseudouridylation level along with evolution (Figure 2).

Since the levels of Ψ modification throughout cancer samples are characterised by a great variability both in the 18S and 28S rRNA, we chose a threshold of log_2_(FC) = ±1 to highlight the most dysregulated positions in tumors with respect to reference RNA samples. These highly modified Ψ sites in our series appeared to be all hypermodified (i.e. having a log_2_(FC) > 1). The list of the highly variable positions occurring in at least 4 out of the 33 (>10%) samples is reported in Supplementary Table S4. In the same table we also show positions in 18S and 28S rRNA displaying an absolute variation of the modification fraction of ±20% compared to the mean of the reference samples, in at least 4 out of the 33 (>10%) samples.

Next, we evaluated the correlation between the expression of specific snoRNAs guiding these relevant pseudouridylation sites and the corresponding pseudouridylation level, comparing with a *t*-test the hypermodified vs not hypermodified samples. For some snoRNAs the expression was considerably higher in the hypermodified samples. However, possibly due to the limited size of the series, it did not result significantly different for any of the considered position (although *P* = 0.057413—was observed for SNORA61 guiding Ψ2495 on 28S—Supplementary Table S4).

### Relationship between rRNA pseudouridylation and tumor features.

In this study, we newly recruited patients with breast cancer that underwent surgery before any chemotherapeutic treatment. This led to the collection of all (but one) luminal cases since standard of care involves neoadjuvant chemotherapy for HER2+ and triple negative breast cancer patients. Bio-pathological features of the included breast cancer cases are summarised in Supplementary Table S5. The relationship between site-specific levels of pseudouridylation and the available data on tumor features is reported in Supplementary Table S6. Strikingly, a negative correlation between many Ψ positions, mainly in 28S rRNA, and age was observed. Furthermore, positions 109 and 119 in 18S and, 4598, 4966 and 4975 in 28S rRNA, were significantly hypermodified in luminal B type tumors with respect to A type ones. This association is also reflected on proliferation, assessed by Ki67 labelling index, which is one of the most important features used to distinguish luminal A to luminal B tumors (32). Specific sites also resulted significantly related to histotype, presence of lymph node metastasis at diagnosis, vascular infiltration of tumor cells, and additional tumor biological features such as p53 accumulation and BCL-2 and EGFR expression.

**Figure 3.**
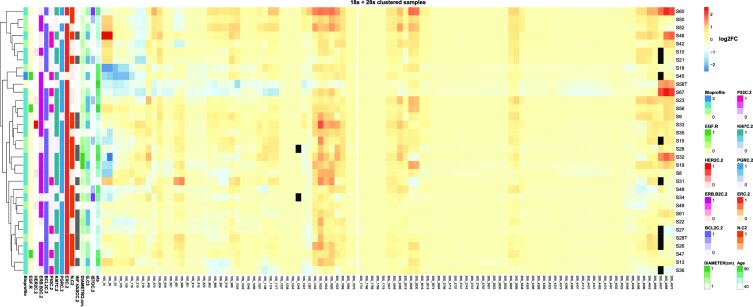
Hierarchical clustering of samples on log_2_ fold-change of known 18S and 28S rRNA pseudouridine positions. The figure shows the log_2_FC of pseudouridine modification levels between tumor samples and the reference RNA samples. Black cells indicate positions for which a quantification of the modification level in that sample was not attainable. Clinical features of the tumor samples are shown on the left side, with the white colour representing unavailable information. Samples hierarchical clustering is presented on the left side.

**Figure 4.**
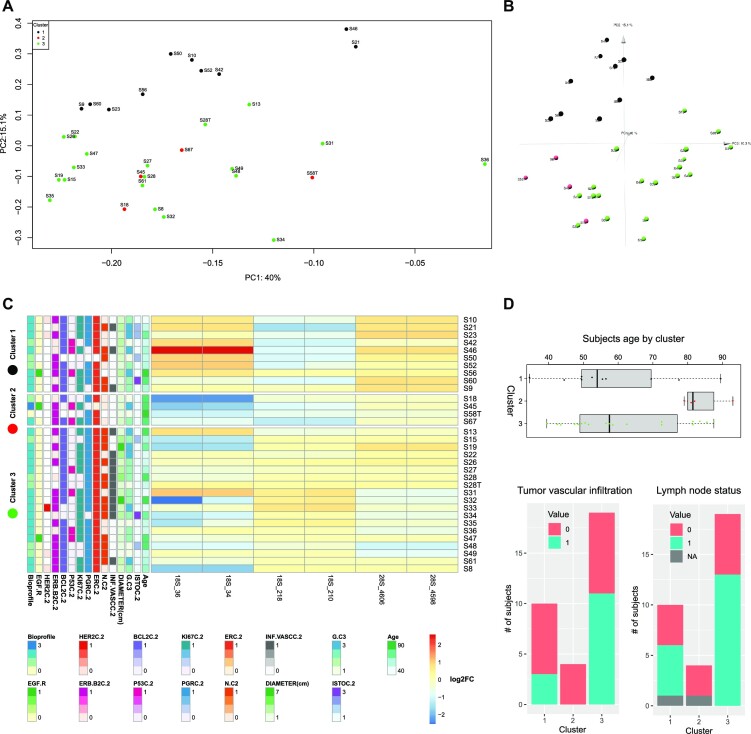
Samples clustering by log_2_FC of Ψ level with respect to reference samples. (**A**) shows the PCA-transformed representation of the samples, using the log2FC of all 18S and 28S positions as features and the first two principal components. Color indicates the cluster to which samples belong, as identified by the hierarchical clustering and k-means algorithms. (**B**) shows the PCA-transformed representation of the samples, using the log2FC of all 18S and 28S positions as features and the first three principal components. Color indicates the cluster to which samples belong, as identified by the hierarchical clustering and *k*-means algorithms. (**C**) shows the log2FC for the positions in the 95th percentile of the highest absolute weight of the PC2 component for the PCA shown above. The log_2_FC values of these positions are able to separate the samples into the three clusters. (**D**) shows the distribution of subjects age, tumor vascular infiltration, and lymph node status for subjects in the three clusters.

We additionally grouped samples of our series by hierarchical clustering according to their pseudouridylation levels on both 18S and 28S rRNAs, also computing the distribution of patients and tumor features (Figure 3). The results indicated the existence of different tumor clusters sharing similar pseudouridylation patterns at specific rRNA regions (namely 18S rRNA positions from 34 to 218; positions from 1445 to 1692; 28S rRNA positions from 1523 to 1564; positions from 1847 to 2830; positions from 3863 to 3938; positions from 4598 to 4975). In particular, the clustering suggested the presence of three major groups (of 7, 23, and 7 samples respectively). To further investigate this potential classification, we performed a k-means clustering analysis with the number of clusters set to 3. The results, plotted through a principal component analysis (PCA) on all 18S and 28S positions as features, substantially confirmed the clusters composition suggested by the hierarchical clustering (Figure 4A). We thus sought to extract the features which contribute most to the definition of these three clusters, and therefore looked at the PC2 axis, which neatly separates clusters 1 and 3. The 95th percentile of the features with the highest weight in this principal component are reported in Figure 4C and belong to two of the above reported regions (18S rRNA 34–218 and 28S 4598–4975). Furthermore, if we include the PC3 axis (Figure 4B), we can also observe the separation of cluster 2 from cluster 1, with specific positions pulling this cluster away from the other two (Supplementary Figure S6). Cluster 2 is characterized by a significantly higher patient age compared to the other two (cluster 2 mean = 83.8 years, cluster 1 mean = 60.2 years, cluster 3 mean = 59.4 years; *P* = 0.02841 (2vs1), 0.02335 (2vs3) - Figure 4D, upper panel). A trend was also observed in the distribution of cases with tumor vascular infiltration (*P* = 0.09317—Figure 4D, lower panel, left) and lymph node metastasis (*P* = 0.05455—Figure 4D, lower panel, right). Considering the limited size of the series and in particular of cluster 2, however, these results should be taken with caution. Furthermore, we also performed a correlation analysis between the snoRNA expression levels we measured via RT-qPCR and the tumor features. The results are reported in Figure S7 and show a few statistically significant correlations, with only some of a sizeable extent (e.g. SNORA67 and SNORA41 with HER2 status).

**Figure 5.**
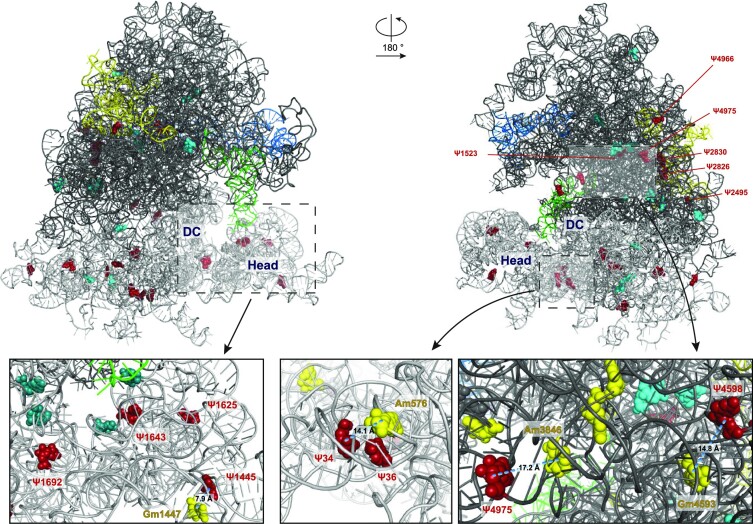
Three-dimensional structure of the human 80S ribosome (obtained from 6QZP.pdb) showing relevant and/or hypermodified pseudouridine sites from two different angles. The 28S rRNA backbone is shown as a dark grey ribbon, the 18S rRNA as a grey ribbon, the 5.8S in yellow, the 5S in blue and the tRNA in light green. Relevant pseudouridine sites (derived from hierarchical sample clustering depicted in Figure 3 and listed in supplementary Table S7) are visualized as cyan spheres; pseudouridine positions derived from PCA-PC2 (showed in Figure 4C) and/or from PCA-PC3 (showed in Figure S6) and/or hypermodified are depicted as red spheres. In the three insets, the modified bases localizing in the decoding centre (DC) are in deepteal green and the 2′-O-methylation sites in close proximity to relevant pseudouridines are in yellow.

Finally, to three-dimensionally locate the pseudouridylation sites that appear to be highly dysregulated or able to differentiate biological clusters of breast cancers (Supplementary Table S7), we mapped them on the human ribosome structure (Figure 5).

## DISCUSSION

The present study provides for the first time a quantitative and site-specific rRNA pseudouridylation profile of human primary tumor samples by means of a bisulfite-based NGS approach, namely RBS-Seq. In particular, a small but homogeneous luminal breast carcinoma series of 33 samples was analysed and the pseudouridylation patterns obtained were correlated to tumor clinical and bio-pathological features.

To perform the pseudouridine profiling, we adapted for rRNA the RBS-seq method, first described for the simultaneous transcriptome-wide detection of m^5^C, Ψ, and m^1^A at single-base resolution. By developing a dedicated pipeline for rRNA analysis we were able to identify almost all known Ψ sites (101/106, 95%), more than in other studies applying bisulfite treatment for pseudouridine detection but with slightly different approaches (27,33). We detected variable pseudouridylation levels at specific sites among tumor samples in comparison to a reference RNA. In this regard it is worth noting that our results highlight a very high variability in absolute uridine modification as compared to what is observed applying different technical approaches (e.g. Mass Spectrometry (13) and HydraPsiSeq (34)), with some positions showing a low absolute modification fraction. These differences can be ascribed to methodological as well as to biological issues. As a technical point, bisulfite may cause the deletion of one or two bases adjacent to the target pseudouridine. However, dealing with rRNA in which pseudouridine may be extremely close to each other, we decided to consider only known positions in rRNA. In this way we might have underestimated the absolute value of modification fractions. In any case since we considered the variation relative to a standard reference RNA we believe that the impact of this specific technical issue in the interpretation of the results has been minimised.

The breast cancer samples from our series were characterised by extensive intra- and inter-tumor variability at each position, both in 18S and 28S rRNA, although they belong to homogeneous tumor types. Specifically, we could identify a number of significantly dysregulated Ψ sites, some of which resulted highly hypermodified. Among them, the most frequent dysregulated site is Ψ1625 in 18S rRNA, being highly modified in 14 samples (41%). This modification site is located in the 18S rRNA Helix 42 (H42), which has been recently reported to be involved in the binding with monovalent cations that are crucial for neck-head interaction in the 40S structure (35). Other recurrently modified positions are represented by Ψ1692 and Ψ1643 on 18S rRNA which stand adjacent on helices H28 and H29, respectively, which also take part in the 40S neck-head junction and are involved in the movement of the tRNA from the P- to the E-site (35). In addition, Ψ1445 on 18S rRNA, also appears to be an interesting site of modification in human breast cancer since it is hypermodified in 4 out of 34 samples. In this regard, we previously observed that increased Ψ1445 modification was associated with the development of neoplastic features in immortalized untransformed mammary gland epithelial cells (29). Interestingly Ψ1445 belong to a group of so- called ‘human-specific’ sites along with Ψ1523, 2495, 2826 and 2830 that we identified as hypermodified on 28S rRNA. These modification sites present a high degree of variability also in human cell lines and samples of human origin detected by the alternative method of HydraPsiSeq (34). Another variable site in 28S rRNA is Ψ4966, which is, conversely, markedly hypomodified in fibroblasts obtained by patients affected by Dyskeratosis congenita (DC), a rare X-linked syndrome caused by mutations in DKC1, the gene encoding for the rRNA pseudouridine synthase (36–38). In this regard, it is interesting to note that some variable 2′-O-Methylation sites identified by Marcel et al. in a larger series of breast cancers (11) are in close proximity to some altered pseudouridylation sites we identified in our study. For instance, 18S-Gm 1447 is adjacent (about 8A) to Ψ1445 (Figure 5, left inset) or 18S-Am 576 which is adjacent (about 14A) to Ψ34 (Figure 5, middle inset). Regarding the large subunit, we could identify a cluster of dysregulated 2′-O-Methylation and Psi sites involving Gm 4588, Gm4590 and Gm4593 and Ψ4598 and Ψ4606 (Figure 5, right inset). Then, without considering the differences between the series analyzed, the results of the two studies are apparently in line in identifying ribosomal domains altered in breast carcinomas. However, it should be noted that while these pseudouridylation sites turned out to be more frequently modified, 2′-O-methylation is instead found decreased. Furthermore, it was previously observed that pseudouridylation at specific sites may influence the modification of adjacent residues (21,22,39,40). Accordingly, we observed that altered pseudouridylation in tumors frequently involve specific structural clusters of pseudouridines (e.g. on 18S rRNA, uridine residues at positions 1445, 1625, 1643 and 1692 are detected as simultaneously hypermodified in 29/33 tumors; similarly, on the 28S, positions 1664, 1670, and 1731 are detected as simultaneously hypomodified in 21/33 tumors - see supplementary Figures S5A and B). A characterization of the structural consequences of this variable regulation is needed by dedicated studies, and will provide mechanistic insights on the effect of these modifications on functional ribosomal domains which can have a role in the cancerous translational machinery. To characterize the origin of the observed site-specific modification variability we evaluated if, for highly variable sites, hypermodification could be linked to correspondent guide snoRNA overexpression. Although our results did not indicate significant associations, a trend could be observed linking the modification of specific sites and the expression of their guide snoRNA. Considering the technical challenges related with snoRNA quantification (41) and the limited size of our series, we believe this issue should be specifically clarified by dedicated studies involving a greater number of samples and the use of unbiased technical approaches for high throughput snoRNA quantification (e.g. TGIRT-seq).

The broad heterogeneity in the degree of rRNA modification we observed for site-specific pseudouridylation is consistent with our previous findings showing a variable level of global pseudouridylation of rRNA in human breast carcinomas (10) and with the data obtained on rRNA 2-’O-methylation in an independent wide series of breast carcinomas (11). In this latter study indeed, two classes of rRNA 2′O-methylation sites were identified upon the variability of the modification levels amongst patients and thus defined as stable or variable sites. Notably, the variable positions are typically human-specific modification sites in comparison to stable positions which are more conserved across evolution (i.e. occurring also in prokaryotes, lower eukaryotes). In this regard, we found a similar pattern for rRNA pseudouridylation as well, where stable sites are the most evolutionary conserved. Altogether, these observations are consistent with a model of a complex evolutionary related system of rRNA modification in which variably modified sites enable the fine-tuning of ribosomal function. On one hand, evolution of eukaryotic rRNAs shows a remarkable phylogenetically-linked expansion via insertions in prokaryote-related core sequences and further enlargement of the inserts (42). On the other hand, rRNA accumulated a number of modification sites, mostly attributable to pseudouridylation and 2′-O-methylation. This could be achieved by the increasing diversity of snoRNAs, due to genetic drift during evolution, that could change their RNA target specificity and even create a new rRNA modification guiding function (43).

Despite the fact that our series is characterised by a relatively limited number of samples with homogeneous characteristics, some clinical and bio-pathological tumor attributes turned out to be significantly associated with pseudouridylation levels at specific sites. This occurs for many modification sites with patients’ age which is also the most different patient feature distinguished by PCA-based sample clustering. This observation is in line with recent studies reporting a link between ribosome activity and ageing (44). rRNA modification is in fact one key factor affecting ribosomal activity (17,45). Our findings cannot however provide information regarding the nature of the association observed, in particular if this reflects a patient feature (i.e. age-related altered modification) or a consequence of the transformation process (i.e. cancer-specific) occurring in a particular subset of patients. Additional observed relationships include cancer cell proliferation, tumor vascular infiltration, and nodal metastasis. Further studies will help to understand if the site-specific modifications associated to these features actually contribute to the development of these important neoplastic characteristics or if they are instead epiphenomena of cancer development. Of note, some of the hypermodified sites mentioned above (i.e. Ψ1692 on 18S rRNA and Ψ4966 and Ψ4975 on 28SrRNA) contribute to distinguish tumors with different features by PCA.

This study represents the first example of the contribution that newly available high-throughput approaches for site specific pseudouridine detection can provide to the understanding of the intrinsic ribosomal changes occurring in human tumors. The data of our series of breast carcinomas exploiting the RBS-Seq technique may be compared in the future with those obtained on different human series, including other subtypes of breast cancer and/or with different technical approaches. In fact, the current availability of Bisulfite-Induced Deletion sequencing (BID)-Seq, HydraPsiSeq and the applications of single molecule sequencing (e.g. nanopore) to pseudouridine detection will likely lead in the near future to a detailed characterization of the alterations involving this specific modification in cancer and possibly in other physiological and pathological conditions (23,33,34,46).

## DATA AVAILABILITY

The RNA-seq datasets generated for this work were deposited in the Gene Expression Omnibus (GEO) with ID GSE220967.

## Supplementary Material

zcad026_Supplemental_Files
