# Supplementary material for: Alterations of ribosomal RNA pseudouridylation in human breast cancer
Source: NAR Cancer. 2023 May 30;5(2):zcad026. doi: 10.1093/narcan/zcad026 (PMC10227372; doi:10.1093/narcan/zcad026)
Supplement: zcad026_Supplemental_Files [file zcad026_supplemental_files.zip › SUPPLEMENTARY DATA Rev_12_05_23.pdf]

SUPPLEMENTARY DATA

SUPPLEMENTARY METHODS

RNA extraction and preparation

Total RNA was extracted from 100-120 mg of frozen specimens of 34 breast tumours using the mirVana miRNA isolation kit (ThermoFisher Scientific) in order to collect transcripts shorter than 200 nts as well. Human total RNA (Human XpressRef Universal Total RNA, Qiagen) prepared from 20 different human adult and foetal normal major organs was used as normal reference samples for analysis. For each tumour sample and the RNA reference sample 5 µg total RNA were used for bisulfite treatment and further steps (RBS sample), while 500 ng total RNA were used as the corresponding non-treated sample (NBS sample). DNAse digestion was carried out with the DNA-free DNA Removal Kit (ThermoFisher Scientific) in a final volume of 20 µl at 37°C for 25 minutes. RNA underwent chemical fragmentation with RNA Fragmentation Reagent (ThermoFisher Scientific) at 70°C for 30 minutes to obtain fragments between 60 and 200 nt. (RNA was quality controlled by 2100 BioAnalyzer using RNA 6000 Nano Kit (Agilent)).

Bisulfite treatment and clean-up

DNAse-digested and fragmented RNA (final volume 45 µl) was denatured in 240 µl of deionized formamide (ThermoFisher Scientific) at 95°C for 5', chilled on ice 10' and 1 µl RNaseOUT (ThermoFisher Scientific) was added. Samples were then incubated with 312 µl of freshly prepared 5M sodium bisulfite (pH 5) and 3 µl of freshly prepared 100mM hydroquinone at 50°C, rotating for 16h. Samples were added with 400 µl RNase-free water and cleaned-up passing through equilibrated Illustra NAP-10 columns (GE Healthcare Life Sciences) and eluted with 1 ml of RNase-free water. Desulfonation was performed splitting the sample into two 0.5ml portions and adding 0.5 ml of 2M Tris buffer pH 9.0 (Trizma preset crystals, Sigma-Aldrich) to each tube, and incubating for 2h at 37°C. RNA was recovered and concentrated by glycogen addition (15µl Glycogen 20 mg/ml (Roche) and 1/10 vol of 3M sodium acetate (pH 5.5)/2.5 vol of ethanol for precipitation at -80°C ON. Samples were centrifuged at 21,000g for 30 min at 4°C. The pellets were then washed once with 1 ml of 70% (vol/vol) ice-cold ethanol, air dried and dissolved in 50 µl of a solution containing 20 mM MgCl2 and 50 mM Tris HCl pH 7. Samples were finally incubated at 75°C for 15' and kept on ice until purification. Bisulfite treated samples and only fragmented non-treated samples were purified using RNAClean XP paramagnetic (Agencourt) beads according to a modified manufacturers' protocol, where the initial step consisted in the addition of 2 volumes isopropanol and 2 volumes of beads to the samples prior to incubation at RT for 10 minutes. RNA was eluted in 20 µl of RNase-free water, quantified by Qubit using the RNA HS Assay and quality-controlled by BioAnalyzer to inspect for the proper fragment length (60-200 nts). RNA samples were stored at -80°C until library preparation.

H/ACA box snoRNAs expression by qRT-PCR

Total RNA of breast cancer specimens used for RBS-seq was subsequently reverse-transcribed using the GOScript kit (Promega) at 55°C following the manufacturer's protocol. Then quantitative real-time PCR (qRT-PCR) with sybr green was assessed to determine the expression of the following H/ACA box snoRNAs: SNORA5A-B; SNORA5C, SNORA22A-B-C; SNORA33, SNORA41, SNORA61, SNORA64, SNORA67,

SNORA70. Primers were designed using the Primer Express software and are listed in Supplementary table S1. qRT-PCR was carried out in triplicate and relative expression levels were calculated using the comparative deltaCt method with beta-actin as house-keeping gene and the reference RNA as calibrator. Real-time PCR analysis was carried out in a 10 µl final volume reaction using 66 ng of cDNA, SsoAdvanced™ Universal SYBR R Green Supermix (Biorad) 2X, 250 nM of each primer in a CFX Connect Instrument (Biorad) with the following amplification parameters: initial denaturation at 95°C for 1 min followed by 40 cycles of denaturation at 95°C for 15 sec and annealing and extension at 60°C for 30 sec.

Bioinformatic and statistical analysis

Reads filtering (minimum base quality of Q30) and adapter trimming was performed with Trim Galore (http://www.bioinformatics.babraham.ac.uk/projects/trim_galore/). Remaining reads were then aligned with meRanTK v1.2.1b (DOI: 10.1093/bioinformatics/btv647) using an index generated with the sequences of 5.8S, 18S, 28S, mt12S, and mt16S rRNAs obtained from the NCBI (https://www.ncbi.nlm.nih.gov/; #U13369.1:6623-6779, X03205.1, U13369.1:7935-12969, respectively.)). The meRanGs mode of meRanTK was used with default parameters. The resulting BAM files of bisulfite-converted (RBS) and untreated samples (NBS) were then used as input for the ScorePseudouridinePosition tool of the RBSSeqTools v1.0 suite (DOI: 10.1073/pnas.1817334116).

SUPPLEMENTARY TABLES LEGEND

Table S1. Sequence and product length of the primers used in qRT-PCR experiments for SNORA expression assessment.

Table S2. Modification fraction (expressed as percentage) of each tumor sample or the mean of two samples and the four replicates reference samples for each pseudouridine known site on 18S and 28S rRNA.

Table S3. List of excluded pseudouridine positions by filtering and statistical analysis and number of samples in which position is excluded.

Table S4. List of the hypermodified pseudouridine sites in 18S and 28S rRNA and number of samples with this feature, together with a list of positions in 18S and 28S rRNA displaying an absolute variation of the modification fraction of +/- 20% compared to the mean of the reference samples, in at least 4 out of the 33 (>10%) samples. The same table also shows the statistics correlation between pseudouridine hyper modification and the expression of the corresponding snoRNA.

Table S5. Table shows biopathological features of breast cancer samples included in this study

Table S6. Table reports the relationship between site-specific levels of pseudouridylation and the available data on tumor features. For continuous variables (age and size) a Pearson correlation test was applied, while for categorical features the t-Student test was performed.

Table S7. The relevant pseudouridine sites for samples clustering, the hypermodified pseudouridine positions and their location in ribosome structure are reported. Positions derived from hierarchical clustering of samples on Log2 fold-change of known 18S and 28S rRNA pseudouridine positions, depicted in Figure 3 are reported in black; positions derived from PCA-PC2 component showed in Figure 4B are in blue; position derived from PCA-PC3 showed in Supplementary Figure S4B are in green.

SUPPLEMENTARY FIGURES LEGEND

FIGURE S1: Pearson correlation matrix of replicated samples. The figure displays the pairwise Pearson correlation of the modification ratio (reads modified/total reads) for each known pseudouridine position in human rRNAs between samples for which a replicate was performed. Darker colour indicates higher correlation, and the number displayed in each cell corresponds to Pearson's r for that pair of samples.

FIGURE S2: Reads per sample normalized by sequence length. The figure shows the normalized number of reads (number of reads / rRNA sequence length) in all sequenced samples. Normalized reads count is shown in red for 18S, black for 5.8S, and blue for 28S.

FIGURE S3 Boxplots representing the modification fraction of pseudouridine sites of bisulfite-treated reference RNA along 18S rRNA (A) and 28S rRNA (B). Pseudouridylation levels are depicted as binned in 5-nucleotide bins minimum and using all available positions, prior to filtering for background levels with the corresponding non-treated (NBS) samples.

FIGURE S4: Psi levels of bisulfite-treated samples at known Psi rRNA sites vs unknown sites (i.e. not previously annotated in rRNA) compared to non-bisulfite-treated samples levels (left) and gradient of Psi levels of nucleotides surrounding known rRNA sites, as detected by deletions in high-throughput sequencing reads (right).

FIGURE S5: Heatmaps showing pseudouridine fraction normalized on reference RNA (expressed as log2(FC)) clustered by hierarchical position in 18S (A) and 28S (B) where in raws are the breast cancer samples, in columns each pseudouridine positions. Black squares: positions excluded by reads filtering analysis as showing a deletion fraction >10% in the NBS or <5% in RBS; red squares: hypermodified positions with log2(FC)>1; * : RBS samples significant positions with respect to NBS and reference RNA.

FIGURE S6: The figure shows the log2FC for the positions in the 95th percentile of the highest absolute weight of the PC3 component for the PCA analysis. The log2FC values of these positions are able to separate samples into the three identified clusters.

FIGURE S7: Correlation analysis (Pearson's for continuous variables such as age and tumor size and point biserial correlation for categorical variables) between the snoRNA expression levels assessed by RT-qPCR and the tumor features for statistically significant pairs. Only correlation values for snoRNA-clinical parameter pairs with a significant p-values are displayed. Non-significant pairs are indicated by “ns”.

FIGURE S1

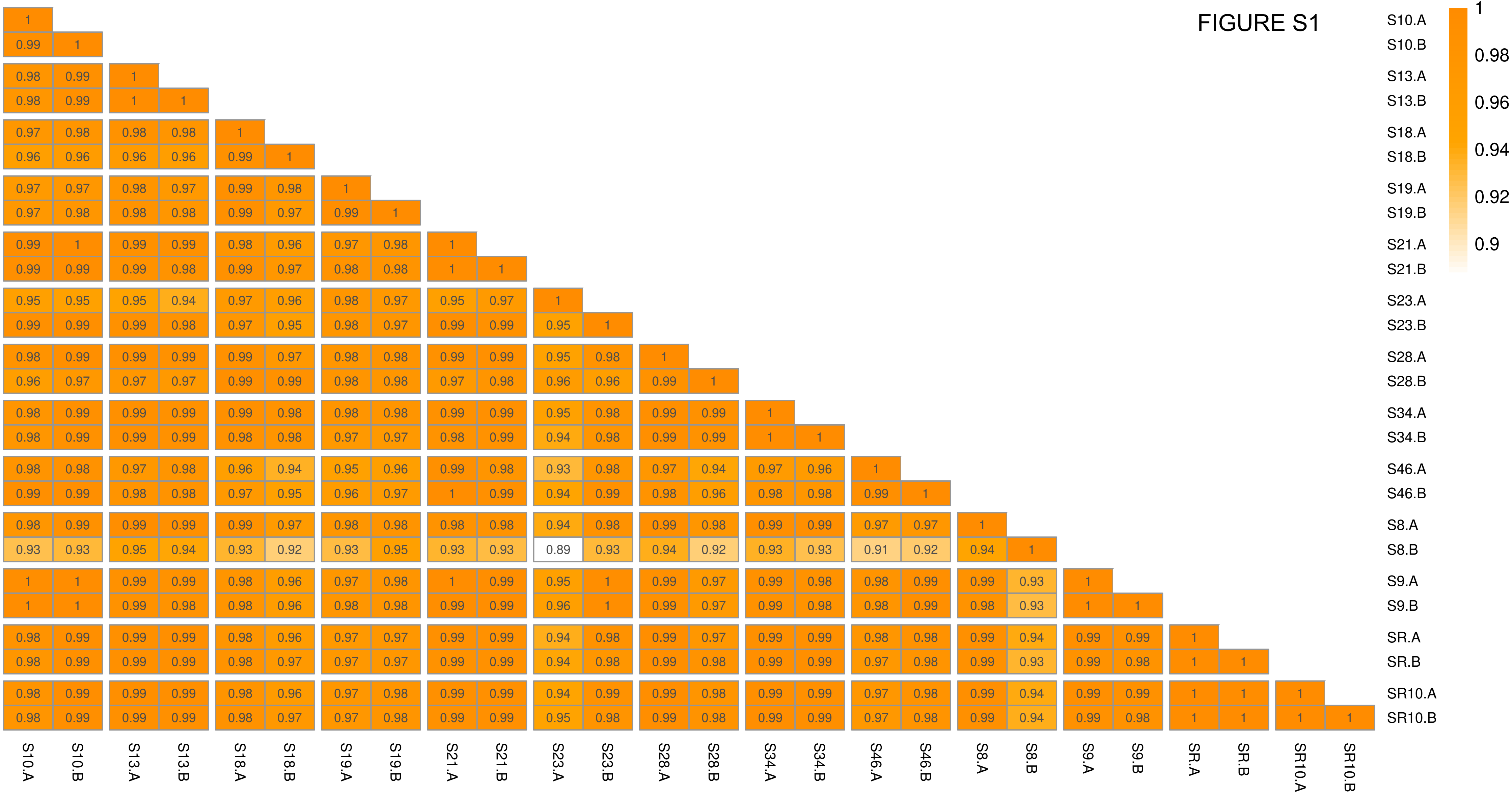
S10.A 1
S10.B 0.99 1
S13.A 0.98 0.99 1
S13.B 0.98 0.99 1 1
S18.A 0.97 0.98 0.98 0.98 1
S18.B 0.96 0.96 0.96 0.96 0.99 1
S19.A 0.97 0.97 0.98 0.97 0.99 0.98 1
S19.B 0.97 0.98 0.98 0.98 0.99 0.97 0.99 1
S21.A 0.99 1 0.99 0.99 0.98 0.96 0.97 0.98 1
S21.B 0.99 0.99 0.99 0.98 0.99 0.97 0.98 0.98 1 1
S23.A 0.95 0.95 0.95 0.94 0.97 0.96 0.98 0.97 0.95 0.97 1
S23.B 0.99 0.99 0.99 0.98 0.97 0.95 0.98 0.97 0.99 0.99 0.95 1
S28.A 0.98 0.99 0.99 0.99 0.99 0.97 0.98 0.98 0.99 0.99 0.95 0.98 1
S28.B 0.96 0.97 0.97 0.97 0.99 0.99 0.98 0.98 0.97 0.98 0.96 0.96 0.99 1
S34.A 0.98 0.99 0.99 0.99 0.99 0.98 0.98 0.98 0.99 0.99 0.95 0.98 0.99 0.99 1
S34.B 0.98 0.99 0.99 0.99 0.98 0.98 0.97 0.97 0.98 0.99 0.94 0.98 0.99 0.99 1 1
S46.A 0.98 0.98 0.97 0.98 0.96 0.94 0.95 0.96 0.99 0.98 0.93 0.98 0.97 0.94 0.97 0.96 1
S46.B 0.99 0.99 0.98 0.98 0.97 0.95 0.96 0.97 1 0.99 0.94 0.99 0.98 0.96 0.98 0.98 0.99 1
S8.A 0.98 0.99 0.99 0.99 0.99 0.97 0.98 0.98 0.98 0.98 0.94 0.98 0.99 0.98 0.99 0.99 0.97 0.97 1
S8.B 0.93 0.93 0.95 0.94 0.93 0.92 0.93 0.95 0.93 0.93 0.89 0.93 0.94 0.92 0.93 0.93 0.91 0.92 0.94 1
S9.A 1 1 0.99 0.99 0.98 0.96 0.97 0.98 1 0.99 0.95 1 0.99 0.97 0.99 0.98 0.98 0.99 0.99 0.93 1
S9.B 1 1 0.99 0.98 0.98 0.96 0.98 0.98 0.99 0.99 0.96 1 0.99 0.97 0.99 0.98 0.98 0.99 0.98 0.93 1 1
SR.A 0.98 0.99 0.99 0.99 0.98 0.96 0.97 0.97 0.99 0.99 0.94 0.98 0.99 0.97 0.99 0.99 0.98 0.98 0.99 0.94 0.99 0.99 1
SR.B 0.98 0.99 0.99 0.99 0.98 0.97 0.97 0.97 0.99 0.99 0.94 0.98 0.99 0.98 0.99 0.99 0.97 0.98 0.99 0.93 0.99 0.98 1 1
SR10.A 0.98 0.99 0.99 0.99 0.98 0.96 0.97 0.98 0.99 0.99 0.94 0.99 0.99 0.98 0.99 0.99 0.97 0.98 0.99 0.94 0.99 0.99 1 1 1
SR10.B 0.98 0.99 0.99 0.99 0.98 0.97 0.97 0.98 0.99 0.99 0.95 0.98 0.99 0.98 0.99 0.99 0.97 0.98 0.99 0.94 0.99 0.98 1 1 1 1
S10.A S10.B S13.A S13.B S18.A S18.B S19.A S19.B S21.A S21.B S23.A S23.B S28.A S28.B S34.A S34.B S46.A S46.B S8.A S8.B S9.A S9.B SR.A SR.B SR10.A SR10.B
1
0.98
0.96
0.94
0.92
0.9

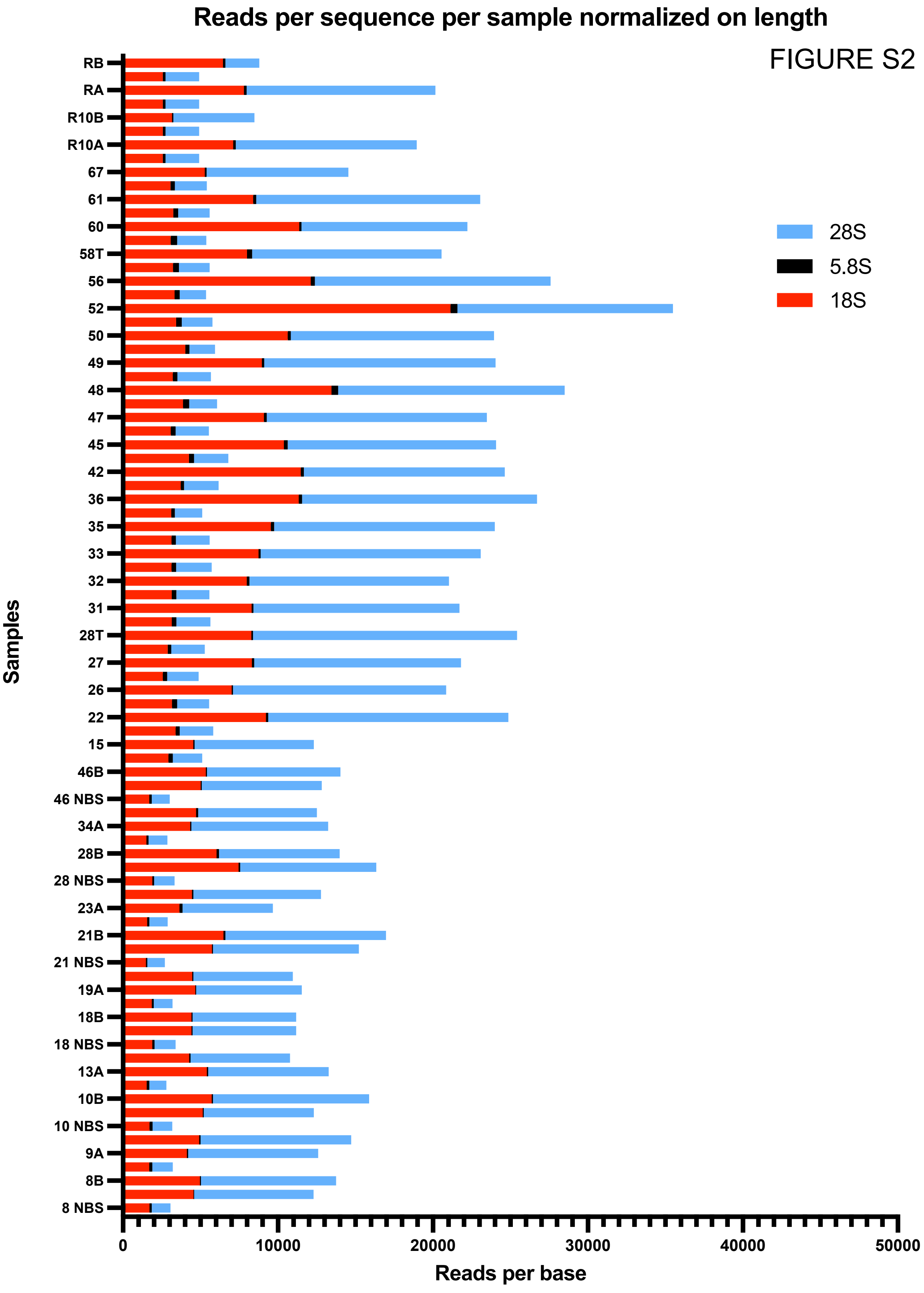

Reads per sequence per sample normalized on length
FIGURE S2
28S
5.8S
18S
RB
RA
R10B
R10A
67
61
60
58T
56
52
50
49
48
47
45
42
36
35
33
32
31
28T
27
26
22
15
46B
46 NBS
34A
28B
28 NBS
23A
21B
21 NBS
19A
18B
18 NBS
13A
10B
10 NBS
9A
8B
8 NBS
Samples
0
10000
20000
30000
40000
50000
Reads per base

FIGURE S3A

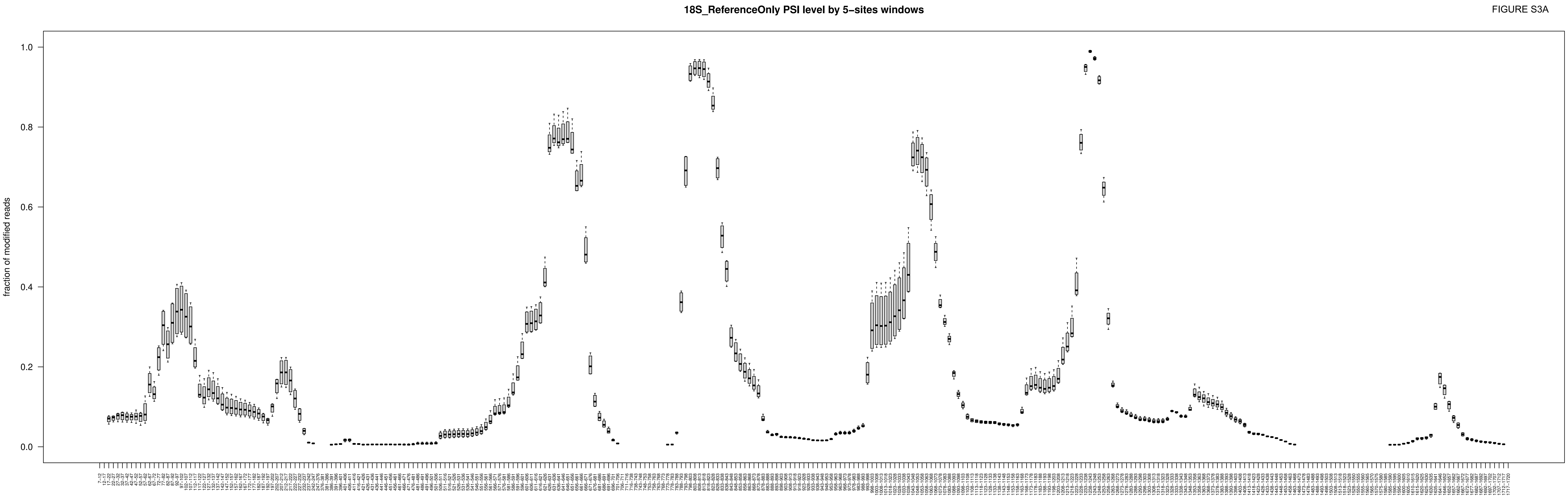
18S_ReferenceOnly PSI level by 5-sites windows
fraction of modified reads
0.0
0.2
0.4
0.6
0.8
1.0

FIGURE S3B

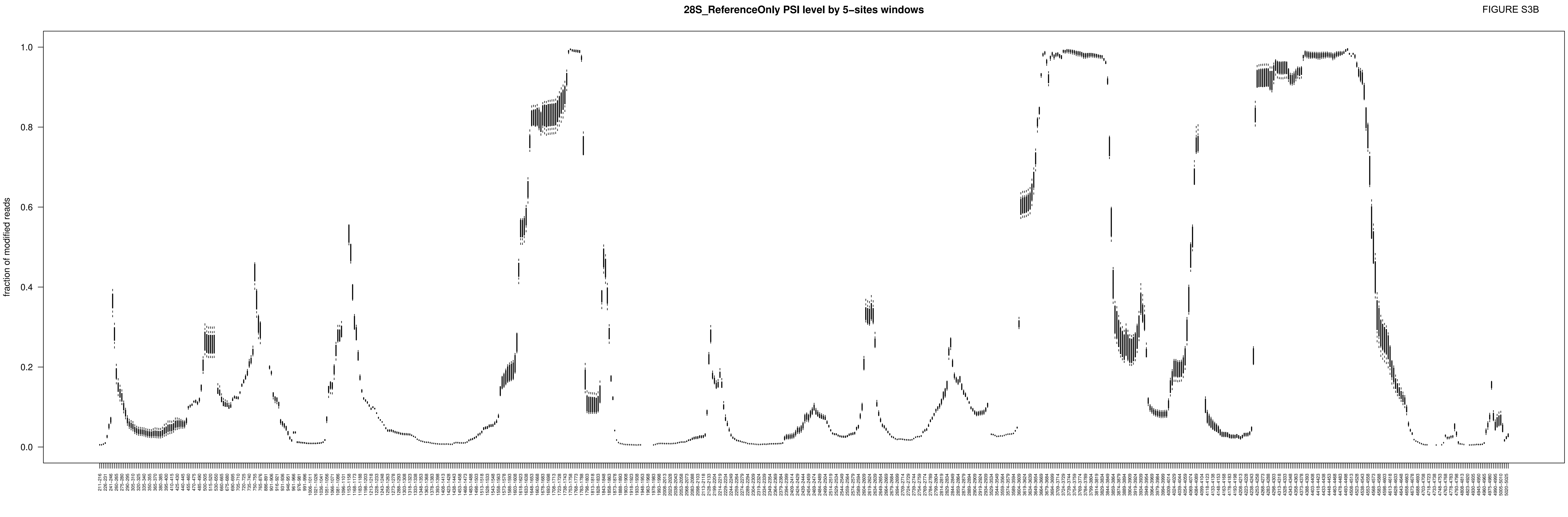
28S_ReferenceOnly PSI level by 5−sites windows
fraction of modified reads
0.0
0.2
0.4
0.6
0.8
1.0

FIGURE S4

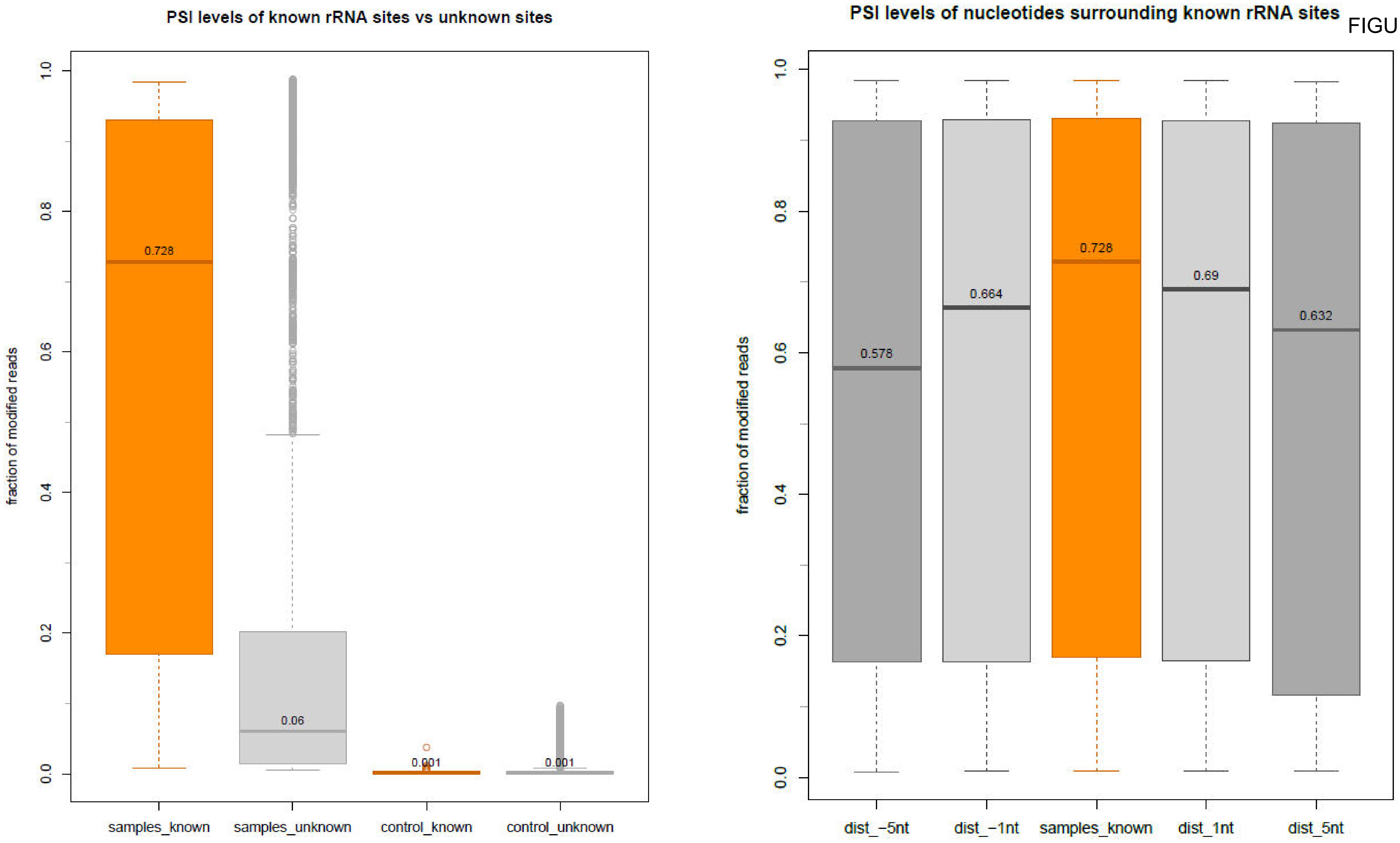

PSI levels of known rRNA sites vs unknown sites
fraction of modified reads
0.0
0.2
0.4
0.6
0.8
1.0
0.728
0.06
0.001
0.001
samples_known
samples_unknown
control_known
control_unknown
PSI levels of nucleotides surrounding known rRNA sites
fraction of modified reads
0.0
0.2
0.4
0.6
0.8
1.0
0.578
0.664
0.728
0.69
0.632
dist_−5nt
dist_−1nt
samples_known
dist_1nt
dist_5nt

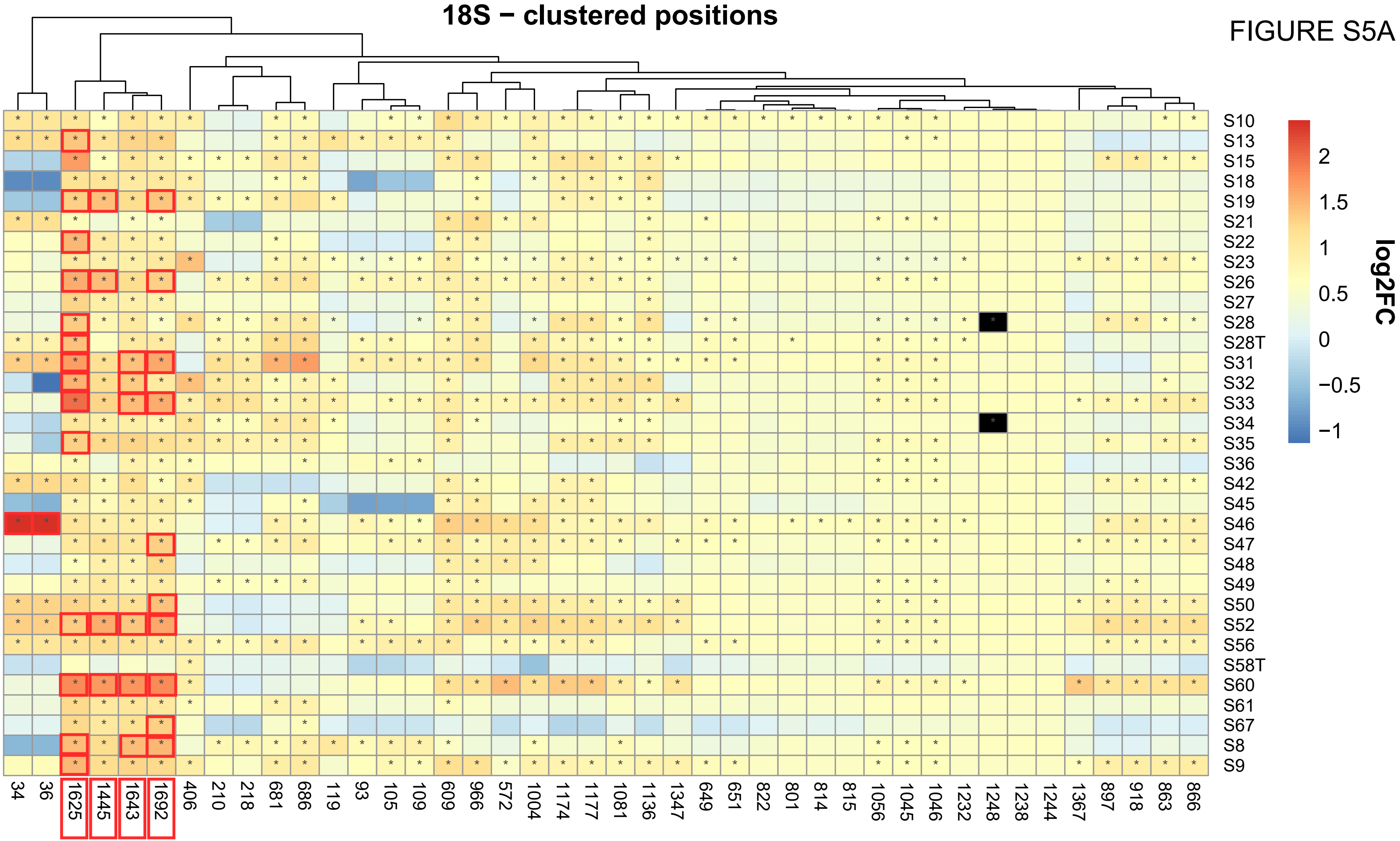
18S − clustered positions
FIGURE S5A
log2FC
2
1.5
1
0.5
0
−0.5
−1
S10
S13
S15
S18
S19
S21
S22
S23
S26
S27
S28
S28T
S31
S32
S33
S34
S35
S36
S42
S45
S46
S47
S48
S49
S50
S52
S56
S58T
S60
S61
S67
S8
S9
34
36
1625
1445
1643
1692
406
210
218
681
686
119
93
105
109
609
966
572
1004
1174
1177
1081
1136
1347
649
651
822
801
814
815
1056
1045
1046
1232
1248
1238
1244
1367
897
918
863
866

FIGURE S5B

28S − clustered positions

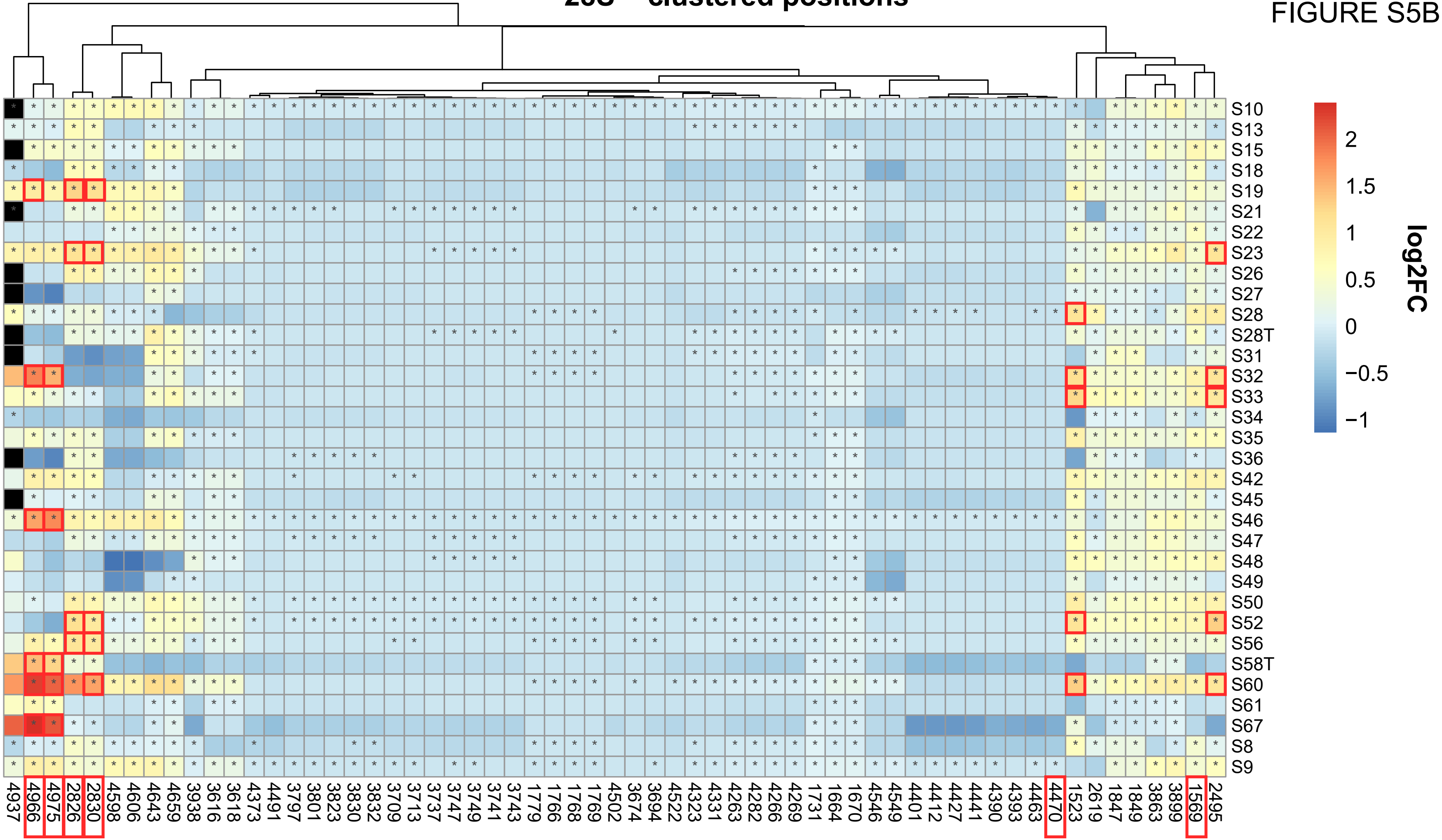

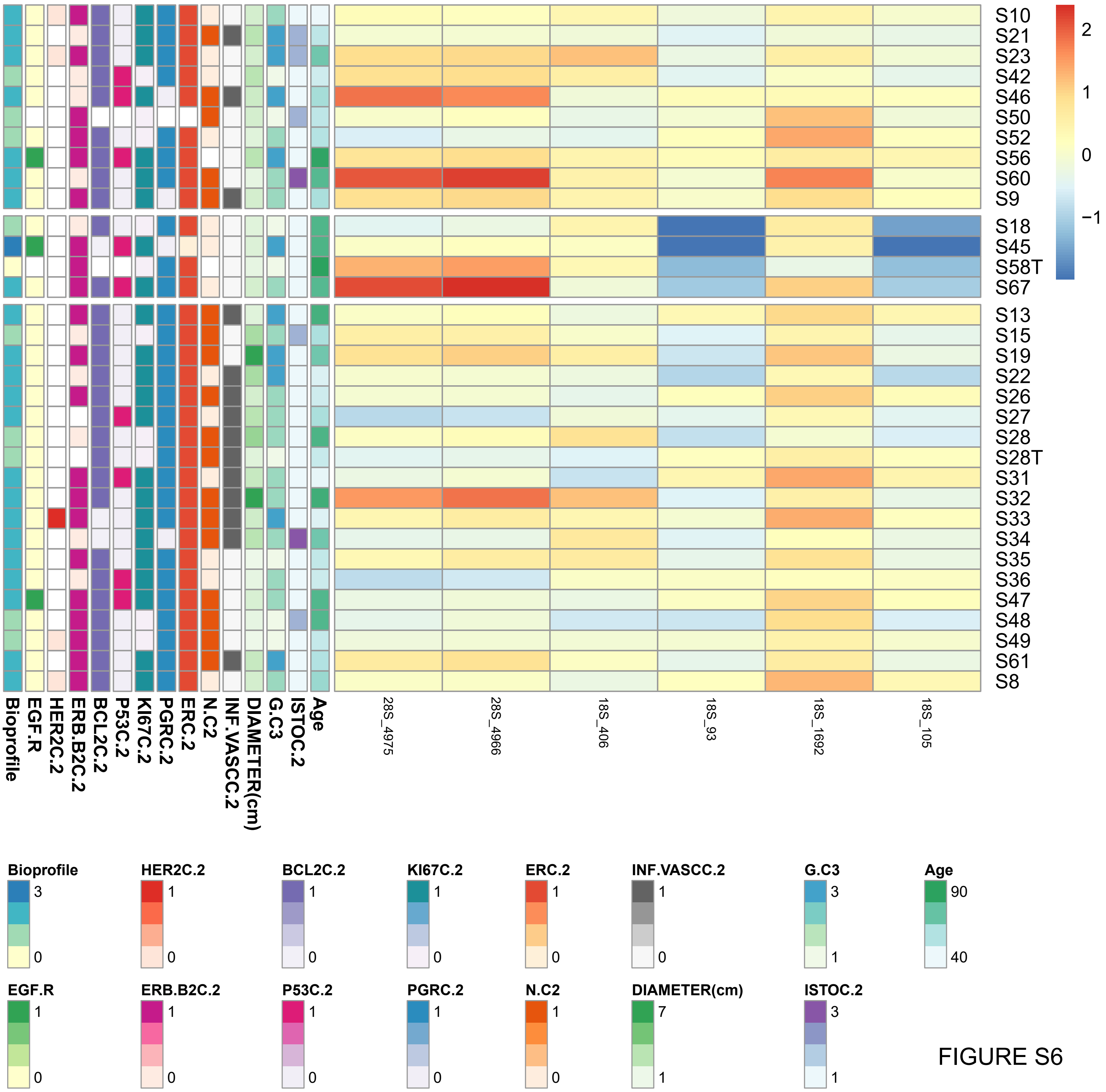
S10
S21
S23
S42
S46
S50
S52
S56
S60
S9
S18
S45
S58T
S67
S13
S15
S19
S22
S26
S27
S28
S28T
S31
S32
S33
S34
S35
S36
S47
S48
S49
S61
S8
2
1
0
−1
Bioprofile
EGF.R
HER2C.2
ERB.B2C.2
BCL2C.2
P53C.2
KI67C.2
PGRC.2
ERC.2
N.C2
INF.VASCC.2
DIAMETER(cm)
G.C3
ISTOC.2
Age
28S_4975
28S_4966
18S_406
18S_93
18S_1692
18S_105
Bioprofile 3 0
HER2C.2 1 0
BCL2C.2 1 0
KI67C.2 1 0
ERC.2 1 0
INF.VASCC.2 1 0
G.C3 3 1
Age 90 40
EGF.R 1 0
ERB.B2C.2 1 0
P53C.2 1 0
PGRC.2 1 0
N.C2 1 0
DIAMETER(cm) 7 1
ISTOC.2 3 1

FIGURE S6

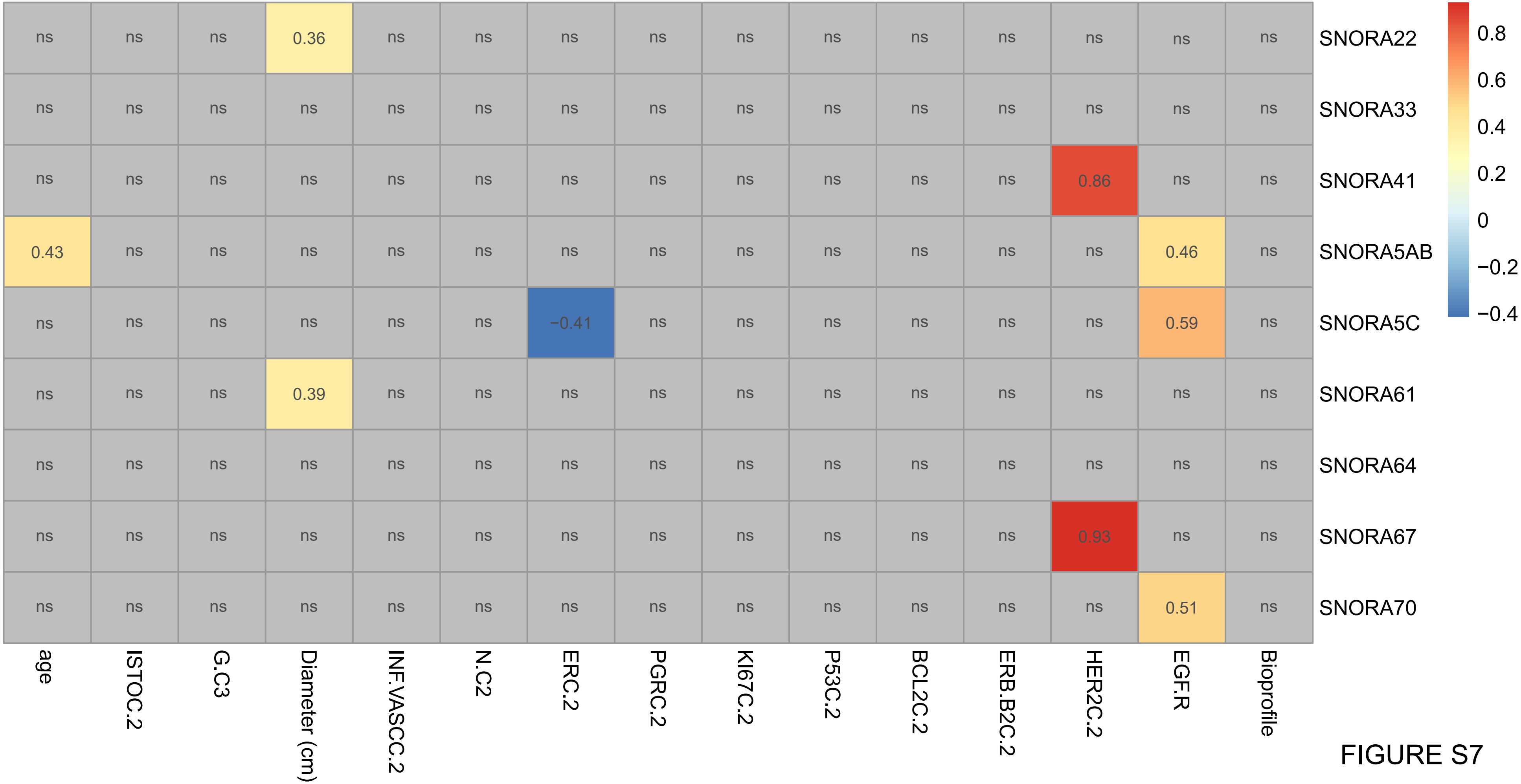
SNORA22
SNORA33
SNORA41
SNORA5AB
SNORA5C
SNORA61
SNORA64
SNORA67
SNORA70
age
ISTOC.2
G.C3
Diameter (cm)
INF.VASCC.2
N.C2
ERC.2
PGRC.2
KI67C.2
P53C.2
BCL2C.2
ERB.B2C.2
HER2C.2
EGF.R
Bioprofile
0.36
0.86
0.43
0.46
−0.41
0.59
0.39
0.93
0.51
ns
0.8
0.6
0.4
0.2
0
−0.2
−0.4

FIGURE S7

TABLE S1 **PRIMERS USED FOR qRT-PCR FOR ASSESSMENT OF SNORA EXPRESSION**

SNORA	Forward	Reverse	Product Size (bp)
SNORA5A-5B	CCGTGTCAAATTCAGTACCTG	CAAATTTATCCCTGAGCCTGG	116
SNORA5C	AGTGCCCGTTTCTGTCATAGC	CAAACTTATCCCCAGGTCCCA	106
SNORA 22A-B-C	AGTTCCCTTGGCTTTGACCC	TGTGCTGGCTTTCTGAATACATTT	101
SNORA33	ACCTGATTGTGTTTGTGCAGA	AGCCATTCTCAGGGACCTTA	102
SNORA41	TGGTAGCAGTTGTGGCATTC	ACCCACTTTCTGTTTTGCTAAGA	80
SNORA61	CTCCTGATCCCTTTCCCATC	CAACATTTAGGCCAGCTTCAC	88
SNORA64	GTGTGACTTTCGTAACGGGGA	TTGCACCCCTCAAGGAAAGAG	91
SNORA67	TCAGGAAAGTAGCAGCTTGGA	CTAAGGAAGGCAGAGGAAAT	85
SNORA70	CCGACTGAGTTCCTTTCCTCA	AGGCTGCGTACACTACCAAG	79
BETA-ACTIN	ATCGTCCACCGCAAATGCTTCTA	AGCCATGCCAATCTCATCTTGTT	105

TABLE S3 **LIST OF EXCLUDED PSEUDOURIDINE POSITIONS BY FILTERING AND STATISTICAL ANALYSIS AND NUMBER OF SAMPLES IN WHICH POSITION IS EXCLUDED**

POSITION	N samples in which position is excluded (33samples+reference)	NOTES
**5.8S**		
**55**	34	not detectable
**69**	34	not detectable
**18S**		
**34**	1	
**36**	2	
**296**	34	not detectable
**681**	1	
**686**	3	
**1232**	5	
**1238**	8	
**1244**	13	
**1248**	34	>10% in the NBS or <5% in RBS sample
**1692**	1	
**28S**		
**3616**	7	
**3618**	6	
**3938**	3	
**4937**	29	>10% in the NBS or <5% in RBS sample

TABLE S4 LIST OF THE HYPERMODIFIED PSEUDOURIDINE SITES IN 18S AND 28S rRNA, NUMBER OF SAMPLES AND CORRELATION WITH CORRESPONDING snoRNA EXPRESSION.

	Ψ	N samples	SNORA	Fold Change HYPERMOD vs not hypermod samples	t-test pval HYPERMOD vs not hypermod samples
18S	1445	4	SNORA67	1,758635	0,421540
	1625	14	SNORA5AB	0,930628	0,851509
	1625	14	SNORA5C	1,010479	0,977438
	1643	6	SNORA41	1,572629	0,267178
	1692	9	SNORA70	0,853689	0,799349
28S	1523	5	Not known		
	2495	5	SNORA61	2,920316	**0,057413**
	2826	5	Not known		
	2830	5	Not known		
	4966	6	SNORA22	1,316424	0,579004
	4966	6	SNORA33	0,833739	0,593795
	4975	5	SNORA64	0,653311	0,191142

LIST OF PSEUDOURIDINE SITES IN 18S AND 28S rRNA WITH +/- 20% DIFFERENCE WITH REFERENCE MEAN MODIFICATION FRACTION, IN AT LEAST 10% OF THE SAMPLES

	Ψ		Ψ
18S	93	28S	1847
	105		1849
	609		3863
	649		3899
	651		4390
	801		4393
	814		4549
	815		4598
	822		4606
	1004		
	1045		
	1046		
	1056		

TABLE S5 BIOPATHOLOGICAL FEATURES OF BREAST CANCER CASES

	Number of patients	(%)
**Total number of patients studied**	34	
**Age (mean +/-SD)**	62,9 +/-16,8	
**Tumor Size (mean +/-SD)**	2,4 +/-1,4	
**Histological Grade**		
1	8	23,5
2	14	41,2
3	12	35,3
**Histological type**		
CDI NST	27	79,4
CLI	5	14,7
CDI NST/CLI	2	5,9
**Vascular infiltration**		
absent	19	55,9
focal/diffuse	15	44,1
**lymph node status**		
N0	14	41,2
N≥1	18	52,9
Not available (NA)	2	5,9
**Estrogen receptor**		
<10%	1	2,9
≥10%	33	97,1
**Progesteron receptor**		
<10%	4	11,8
≥10%	30	88,2
**Proliferation index (by Ki67)**		
<20%	11	32,4
≥20%	23	67,6
**p53 mutation**		
<10%	24	70,6
≥10%	10	29,4
**Bcl2 staining**		
negative	3	8,8
intermediate/positive	31	91,2
**ErbB2 staining**		
negative	9	26,5
positive	23	67,6
NA	2	5,9
**HER2**		
non amplification	6	17,6
amplification	1	2,9
NA	27	79,4
**EGFR staining**		
negative	31	91,2
positive	2	5,9
NA	1	2,9
**Molecular type**		
lum A	10	29,4
lum B	23	67,6
TNBC	1	2,9

TABLE S7 **RELEVANT PSI FOR SAMPLES CLUSTERING, HYPERMODIFIED PSI AND LOCATION IN RIBOSOME**

	Ψ	PCA-PC2	PCA-PC3	hypermodified respect to Reference	Location in ribosome
**18S**	34	34			h4
	36	36			h4
	93		93		h7
	105		105		h7
	109				h7
	119				h7
	210	210			h9
	218	218			h9
	406		406		h11
	686	686	686		
	1445			1445	h34
	1625			1625	h42
	1643			1643	h29
	1692		1692	1692	h28
**28S**	1523			1523	h33
	1569				h34
	1847				h39
	1849				h39
	2495			2495	h52
	2619				h57
	2826			2826	h61
	2830			2830	h61
	3863				h72
	3899				h74
	3938				h76
	4598	4598			h96
	4606	4606			h96
	4643				h96
	4659				h97
	4937				h100
	4966		4966	4966	h101
	4975		4975	4975	h101

black samples clustering (FIG. 3)
blue PCA-PC2 clustering (PCA- PC2 FIG 4B)
green PCA-PC3 clustering (PCA- PC3 FIG S4B)
